# Evaluation of intracellular processes in quinolinic acid-induced brain damage by imaging reactive oxygen species generation and mitochondrial complex I activity

**DOI:** 10.1186/s13550-021-00841-3

**Published:** 2021-10-09

**Authors:** Rie Hosoi, Yuka Fujii, Ohba Hiroyuki, Miho Shukuri, Shingo Nishiyama, Masakatsu Kanazawa, Kenichiro Todoroki, Yasushi Arano, Toshihiro Sakai, Hideo Tsukada, Osamu Inoue

**Affiliations:** 1 Division of Health Sciences, Graduate School of Medicine, Osaka University, 1-7 Yamadaoka, Suita, Osaka 565-0871 Japan; 2 Central Research Laboratory, Hamamatsu Photonics K. K, 5000 Hirakuchi, Hamakita, Hamamatsu, Shizuoka 434-8601 Japan; 3 Laboratory of Physical Chemistry, Showa Pharmaceutical University, 3-3165 Higashi-Tamagawagakuen, Machida, Tokyo 194-8543 Japan; 4 Department of Analytical and Bio-Analytical Chemistry, School of Pharmaceutical Sciences, University of Shizuoka, 52-1 Yada, Suruga-ku, Shizuoka, Shizuoka 422-8526 Japan; 5 Graduate School of Pharmaceutical Sciences, Chiba University, 1-8-1 Inohana, Chuo-ku, Chiba, Chiba 260-8675 Japan; 6 Hanwa Intelligent Medical Center, Hanwa Daini Senboku Hospital, 3176 Fukaikitamachi, Naka-ku, Sakai, Osaka 599-8271 Japan

**Keywords:** Reactive oxygen species, Mitochondrial complex I, Dihydroethidium, ^18^F-BCPP-EF, Quinolinic acid, Microglia

## Abstract

**Purpose:**

Our study aimed to elucidate the intracellular processes associated with quinolinic acid (QA)-induced brain injury by acquiring semiquantitative fluorescent images of reactive oxygen species (ROS) generation and positron emission tomography (PET) images of mitochondrial complex I (MC-I) activity.

**Methods:**

Ex vivo fluorescent imaging with dihydroethidium (DHE) and PET scans with ^18^F-BCPP-EF were conducted at 3 h and 24 h after QA injection into the rat striatum. Immunohistochemical studies were performed 24 h after QA injection into the rat brain using monoclonal antibodies against neuronal nuclei (NeuN) and CD11b.

**Results:**

A strong DHE-derived fluorescent signal was detected in a focal area within the QA-injected striatum 3 h after QA injection, and increased fluorescent signal spread throughout the striatum and parts of the cerebral cortex after 24 h. By contrast, ^18^F-BCPP-EF uptake in the QA-injected rat brain was unchanged after 3 h and markedly decreased after 24 h, not only in the striatum but also in the cerebral hemisphere. The fluorescent signal in the striatum 24 h after QA injection colocalised with microglial marker expression.

**Conclusions:**

We successfully obtained functional images of focal ROS generation during the early period of excitotoxic injury, and microglial ROS generation and mitochondrial dysfunction were observed during the progression of the inflammatory response. Both ex vivo DHE imaging and in vivo ^18^F-BCPP-EF-PET were sufficiently sensitive to detect the respective processes of QA-induced brain damage. Our study contributes to the functional imaging of multiple events during the pathological process.

**Supplementary Information:**

The online version contains supplementary material available at 10.1186/s13550-021-00841-3.

## Introduction

In vivo and ex vivo brain imaging has provided useful information for the study of brain damage and dysfunction. Quantitative imaging of the living brain is useful for elucidating the processes underlying neurodegenerative diseases. The overproduction of reactive oxygen species (ROS) and mitochondrial dysfunction play important roles during the process of brain damage and dysfunction. Our study aimed to provide semi-quantitative imaging information of these two intracellular functions in the living brain, both in the early stages of excitotoxicity and in the progression of the inflammatory response. Oxidative stress is closely associated with mitochondrial dysfunction, and mitochondria are both the major sources and primary targets of ROS. Mitochondrial complex I (MC-I), in addition to MC-III, is considered to be the primary site of ROS generation [1, 2]. ROS play an important role as a regulatory mediator of signalling processes under physiological conditions, and relatively low ROS concentrations are maintained by the redox regulation system [3]. However, the persistent production of excessive ROS induces a disturbance in redox homeostasis. Excessive amounts of ROS cause oxidative damage to DNA, proteins, and lipids, resulting in cellular damage and cell death. Therefore, ROS and mitochondrial dysfunction have been implicated in many neurodegenerative disorders, including Alzheimer’s disease; Parkinson’s disease; multiple sclerosis; cerebrovascular diseases, such as ischaemia/reperfusion injury; and several psychiatric disorders [4–11].

Dihydroethidium (DHE) is a lipophilic membrane-permeable compound converted into charged membrane-impermeable products by superoxide radicals. When DHE products intercalate within the DNA, the nucleus becomes stained with a bright fluorescent red [12]. When systemically administered to animals, DHE distributes rapidly into various tissues, including the brain; if not oxidised, DHE is cleared from tissues and excreted. DHE has been widely used to evaluate ROS production in both in vitro and in vivo experiments [13, 14]. Recently, we reported a simple procedure for imaging excessive ROS generation in intact animals utilising a planar laser scanner, Fluoro-Imaging Analyzer System (FLA-7000; Fuji Film Co, Tokyo, Japan), and a fluorescent probe, DHE. In the previous report, we successfully obtained semiquantitative fluorescent images of ROS overexpression in brain and renal disorders [15]. In this study, we applied this simple procedure to both the early stages of excitotoxicity and the progression of the inflammatory response in living animals.

For quantitative imaging of MC-I activity in the living animal brain, we previously developed a positron emission tomography (PET) probe, 2-tert-butyl-4-chloro-5-{6-[2-(2-^18^F-fluoroethoxy)-ethoxy]pyridin-3-ylmethoxy}-2H-pyridazin-3-one (^18^F-BCPP-EF) [16], and evaluated its ability to detect age-related reductions in MC-I activity in the brains of monkeys [17] and humans [18]; neurodegenerative damage after ischaemia–reperfusion injury in the brains of live rats [19] and monkeys [20]; and Alzheimer’s disease-related reductions in MC-I activity in the brains of monkeys [21] and humans [22]. 2-Deoxy-2-[^18^F]fluoro-glucose (^18^F-FDG) is a commonly used probe for the quantitative measurement of glucose metabolism; however, ^18^F-FDG is also taken up by activated inflammatory cells (microglia and macrophages) [20, 23, 24]. In contrast, ^18^F-BCPP-EF demonstrated reduced uptake in the regions associated with high immunoreactivity against ionised calcium-binding adaptor molecule 1 (Iba1), a microglial marker and was well-correlated with the regional cerebral metabolism of oxygen (rCMRO_2_) in the ischaemic animal brain [20]. ^18^F-BCPP-EF is expected to better reflect mitochondrial dysfunction and cellular energy deficiencies in neurons than ^18^F-FDG.

In the present study, we evaluated the intracellular processes that occur in the quinolinic acid (QA)-treated rat brain: ROS generation and MC-I activity. QA is an endogenous metabolite of the kynurenine pathway that acts as an N-methyl-D-aspartate (NMDA) receptor agonist [25, 26]. QA has a potent neurotoxic effect, and the intrastriatal injection of QA in the rat brain has been used to obtain an excitotoxic model of Huntington’s disease [27–29]. There have been no previous reports of imaging of excessive ROS generation or mitochondrial dysfunction in the QA-injected living rat brain. During the present study, we obtained functional images of the focal ROS generation 3 h after QA injection, and ROS generation in microglia and mitochondrial dysfunction were imaged 24 h after QA injection.

## Materials and methods

### Animals and chemicals

Adult male Sprague Dawley rats (8–9 weeks old) were obtained from CLEA Japan, Inc. (Tokyo, Japan). Animals were housed on a 12-h light/dark cycle with free access to food and water. The following animal experiments were approved by the Institutional Animal Care and Use Committee, Division of Health Sciences, Graduate School of Medicine, Osaka University (Approval numbers; 31–02-2) and the Ethics Committee of the Central Research Laboratory, Hamamatsu Photonics (Approval numbers; HPK-2019-07B).

**Fig. 1:**
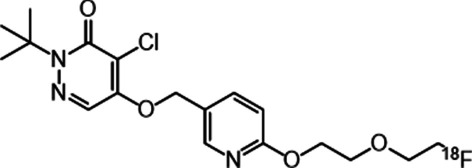
Structures of ^18^F-BCPP-EF

2,7-Diamino-10-ethyl-9-phenyl-9,10-dihydrophenanthridine (DHE) was obtained from AdipoGen AG (Liestal, Switzerland). 2,3-Pyridinedicarboxylic acid (QA) was obtained from Nacalai Tesque Inc. (Kyoto, Japan). The precursor of 2-tert-butyl-4-chloro-5-{6-[2-(2-18F-fluoroethoxy)-ethoxy]pyridin-3-ylmethoxy}-2H-pyridazin-3-one (^18^F-BCPP-EF, Fig. 1) and its corresponding cold compounds were purchased from NARD Institute (Amagasaki, Japan). The synthesis of ^18^F-BCPP-EF was performed as described previously [16]. Radiochemical yields, radiochemical purities, and specific radioactivity of ^18^F-BCPP-EF was 44%, 100%, and 27.7 GBq/μmol, respectively. All other chemicals utilised in this study were the highest grade commercially available.

### Surgery and microinjection of QA

Surgery preparation and QA injections were performed according to previously described protocols [30–33]. For the implantation of guide cannulas into the striatum, the animal was anaesthetised with isoflurane (induction 5%, maintenance 2%). Next, the head of the animal was placed in a stereotaxic apparatus. Bilateral 26-gauge stainless steel guide cannulas fitted with 30-gauge stainless steel obturators were placed into the striatum at the following position: 3.2 mm lateral to the midline, 0.2 mm anterior to Bregma, and 2.0 mm below the cortical surface [34]. The guide cannulas were then fixed to the skull with acrylic cement and two stainless steel screws. After the surgery, the animals were allowed to recover for several days.

QA (60 nmol/µl) was dissolved in saline, and the drug solution was injected (0.2 µl/min, 10 min) through a 30-gauge cannula (5.5 mm below the cortical surface) into the left striatum of each rat while the rat was awake. The injection cannula was left in place for an additional 3 min to reduce the reflux of the injected chemicals along the cannula track. Simultaneously, saline solution was injected through a cannula (30-gauge, 5.5 mm below the cortical surface) into the right striatum. Subsequently, animals were subjected to ex vivo DHE experiments and PET measurements using ^18^F-BCPP-EF.

### DHE administration and ROS detection with the FLA-7000

DHE (5 mg/kg) was administered through the tail vein 3 h and 24 h after the QA injection. The animals were then sacrificed 60 min after the injection of DHE by decapitation under brief anaesthesia. The brains were quickly removed and frozen. Next, coronal slices (20-µm-thick) were prepared using a cryostat at –20 °C and placed onto glass slides. The brain slices were scanned using a 532 nm excitation laser and a 580 nm long-pass detection filter with a Fluoro-Imaging Analyzer System (FLA-7000; Fuji Film Co., Tokyo, Japan). The fluorescence intensity in the brain slices was measured in linear arbitrary units (LAU), corrected for background ([LAU-background] / area [mm^2^]) with Multi-Gauge Analysis Software (Fuji Film Co., Tokyo, Japan). The background value was obtained as the fluorescence intensity of a region of the glass slide containing no brain slices.

### PET imaging

^18^F-BCPP-EF (58.5–66.7 MBq, 2.7–3.7 nmol/kg animal, iv) was injected 3 h and 24 h after the QA injection. The PET scans were conducted with a high-resolution animal PET scanner (SHR-38000; Hamamatsu Photonics, Hamamatsu, Japan) under isoflurane anaesthesia. Dynamic images and summation images of ^18^F-BCPP-EF, from 0 to 90 min after the injection, were reconstructed and used to create standardised uptake value (SUV) images. The body temperatures of the rats were monitored and maintained using a heating pad during PET measurement. Volumes of interest (VOIs) were placed on PET images in the striatum, prefrontal cortex, frontal cortex, parietal cortex, hippocampus, thalamus, and cerebellum, with the aid of Waxholm Space Atlas of the Sprague Dawley Rat Brain (the INCF Software Center, https://www.nitrc.org/projects/whs-sd-atlas) and rat brain atlas [34].

### Histochemistry

The frozen sections used in the ex vivo DHE experiments were stained with Cresyl violet. The stained sections were then visualised using a Nikon Eclipse 80i microscope (Nikon Co., Tokyo, Japan), and the images were captured using the NIS-Elements BR 2.30 software (Nikon Co., Tokyo, Japan).

For the immunohistochemical study, the rats were deeply anaesthetised and perfused transcardially with ice-cold saline, followed by 4% paraformaldehyde solution, 24 h after QA injection. The brains were removed, post-fixed with paraformaldehyde solution for 6 h, placed in a 30% sucrose solution, and embedded in optimal cutting temperature (OCT) compound (Sakura Finetek Japan Co., Ltd., Tokyo, Japan). Serial coronal Sects. (20-µm-thick) were obtained with a cryostat and thaw-mounted on a glass slide. The antibodies used in this study were as follows: mouse monoclonal anti-neuron-specific nuclear protein (NeuN, 1:500, Merck Millipore Co., Darmstadt, Germany), and mouse monoclonal anti-CD11b antibody (clone OX-42, 1:500, AbD Serotec, Oxfordshire, UK). After the overnight incubation of the brain sections with the antibodies at room temperature, the primary antibodies were visualised with Cy2-labelled secondary antibody (1:500, Jackson ImmunoResearch, Newmarket, UK). Fluorescent images were captured with a 3- laser confocal microscope (Nikon Co., Tokyo, Japan). The number of cells with each marker in the microscopic field of view of the rat striatum was counted.

### Statistical analysis

All values are expressed as the mean ± SD (for each group). Differences were assessed with Student’s paired t-tests. P-values less than 0.05 were considered significant.

## Results

**Fig. 2:**
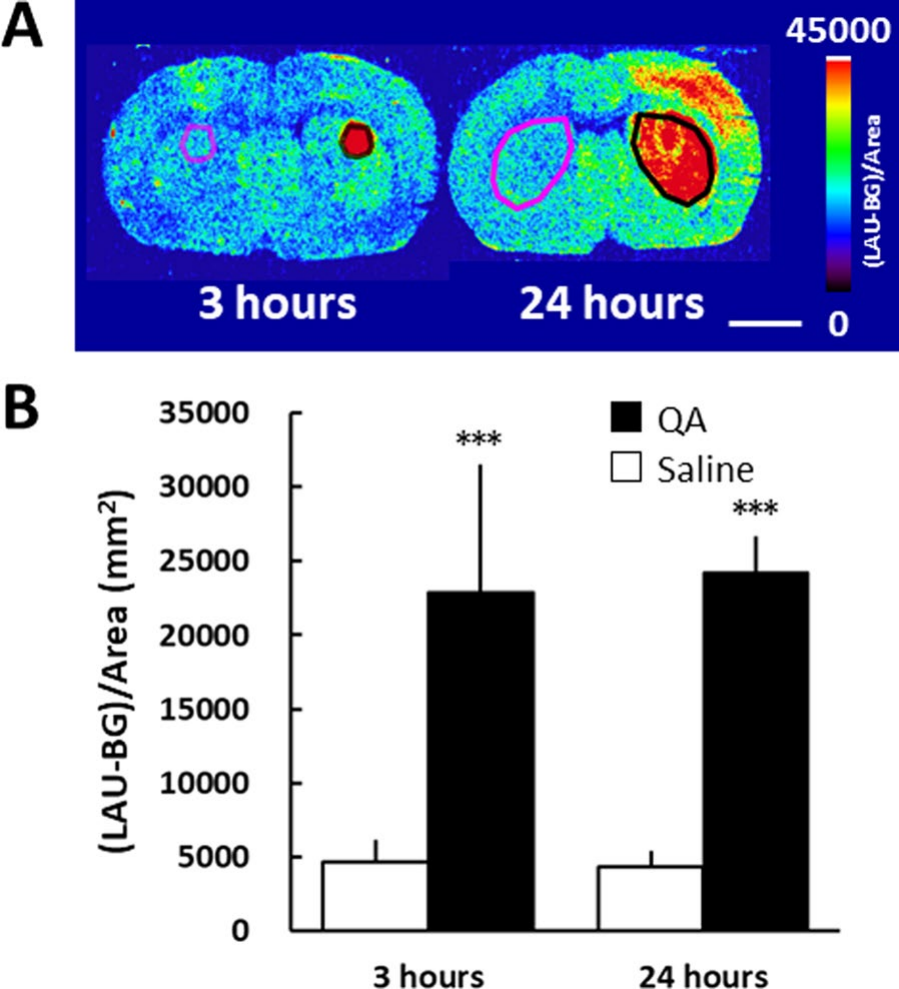
DHE uptake at 3 h and 24 h after QA injection. QA was injected into the striatum on the right side of the image, and saline was simultaneously injected into the contralateral striatum. **A** Accumulation of fluorescence in the QA-injected striatum 60 min after the intravenous injection of DHE. **B** Semiquantitative analysis of the fluorescent images. The regions of interest (ROIs) were identified based on the strong fluorescence signal region of the striatum and the corresponding region on the opposite side. Mean ± SD, n = 4–9. ***P < 0.001, between the saline- and the QA-injected side using the Student’s paired t-test. Bar = 5 mm

A typical distribution of QA-induced fluorescent signal in the rat brain slices at the striatal level is shown in Fig. 2A. A focal strong fluorescent signal in a small area and a slight increase in fluorescent signal throughout the striatum were detected in the QA-injected side 3 h after the QA injection, and the strong fluorescent signal had spread throughout the entire striatum by 24 h after QA injection (Fig. 2A and B). A marked increase in the fluorescent signal was also detected in specific regions of the cerebral cortex 24 h after QA injection, as shown in the right panel of Fig. 2A.

**Fig. 3:**
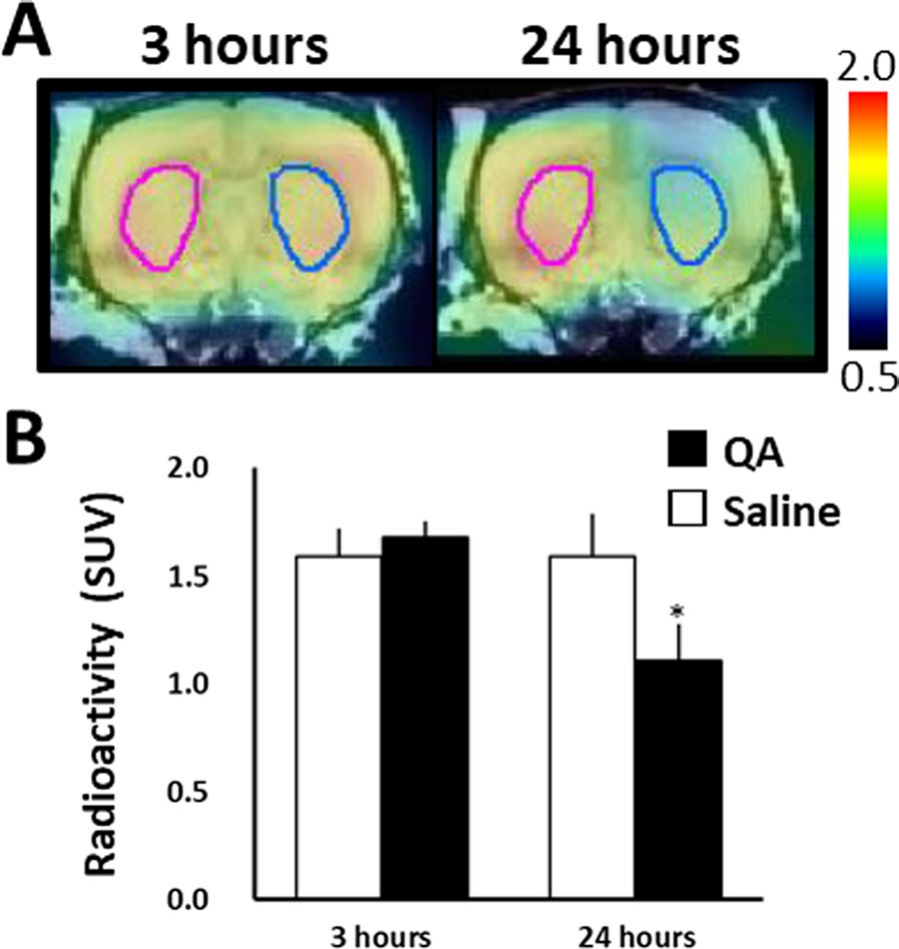
^18^F-BCPP-EF uptake at 3 h and 24 h after QA injection. QA was injected into the striatum on the right side of the image, and saline was simultaneously injected into the contralateral striatum. **A** PET images of ^18^F-BCPP-EF uptake in QA-injected rat brains were superimposed on the MRI atlas images. PET scans were performed for 90 min with ^18^F-BCPP-EF, 3 h and 24 h after QA injection. Summation PET images from 45 to 60 min were reconstructed to obtain standardised uptake value (SUV) images. **B** Radioactivity concentrations for the time period from 45–60 min (SUV). The VOIs were identified based on the whole striatum. Mean ± SD, n = 4. *P < 0.05, between the saline- and QA-injected sides using the Student’s paired t-test

For the evaluation of QA injections effects on MC-I activity in the brain, we obtained PET images using ^18^F-BCPP-EF at 3 h and 24 h after QA injection (Fig. 3A). Three hours after QA injection, no significant differences in ^18^F-BCPP-EF uptake were observed between saline- and QA-injected brain hemispheres. By contrast, 24 h after QA injection, ^18^F-BCPP-EF uptake was significantly decreased by 30.9% compared with that on the saline-injected side (Fig. 3B) throughout the whole striatum.

**Fig. 4:**
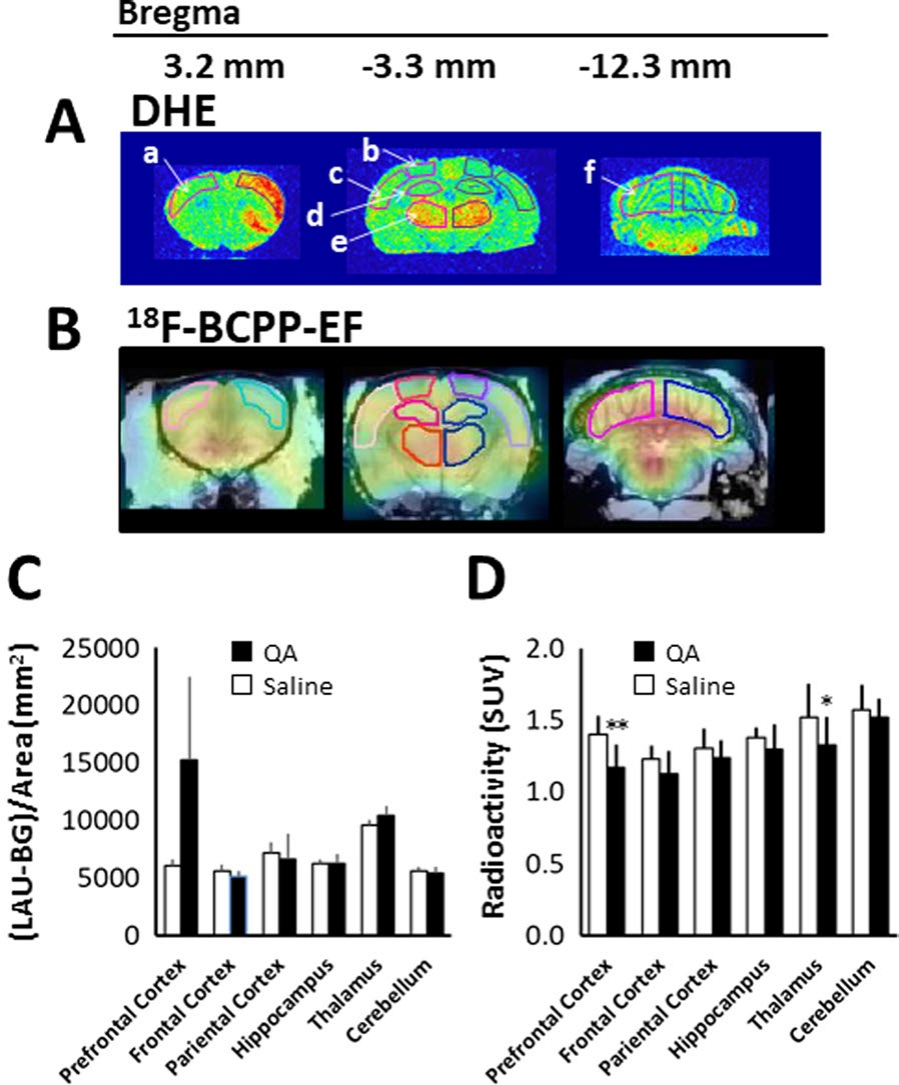
DHE and ^18^F-BCPP-EF uptake in various brain regions 24 h after QA injection. QA was injected into the striatum on the right side of the image, and saline was simultaneously injected into the contralateral striatum. **A** The accumulation of fluorescence in the QA-injected striatum 60 min after the intravenous injection of DHE. **B** Summation ^18^F-BCPP-EF PET images from 45 to 60 min were reconstructed to obtain standardised uptake value (SUV) images. **C** Semiquantitative analysis of the fluorescent images. The ROIs were identified based on the prefrontal cortex (a), frontal cortex (b), parietal cortex (c), hippocampus (d), thalamus (e), and cerebellum (f). Mean ± SD, n = 4. D; Radioactivity concentration for the time period from 45–60 min (SUV). The VOIs were identified based on the prefrontal cortex, frontal cortex, parietal cortex, hippocampus, thalamus, and cerebellum. Mean ± SD, n = 4. *P < 0.05, **P < 0.01 between the saline- and the QA-injected side using the Student’s paired t-test

The fluorescence signals from DHE-injected rats tended to increase in the prefrontal cortex 24 h after QA injection, but no changes were observed in other regions (Fig. 4A and C). By contrast, ^18^F-BCPP-EF uptake 24 h after QA injection was reduced not only in the striatum but also in cerebral regions. Thus, ^18^F-BCPP-EF uptake was significantly reduced in the prefrontal cortex and thalamus. Although no significant difference was observed in the frontal cortex, parietal cortex, and hippocampus, a tendency towards reduced ^18^F-BCPP-EF uptake was observed following QA injection (Fig. 4B and D), indicating that the QA-induced impairment of MC-I activity expanded from the injection point into surrounding areas. When comparing the extent of QA influence 24 h after injection between the DHE-derived fluorescent signal and ^18^F-BCPP-EF uptake, we found that ^18^F-BCPP-EF uptake was more strongly affected by QA over a larger area of the cerebral hemisphere.

**Fig. 5:**
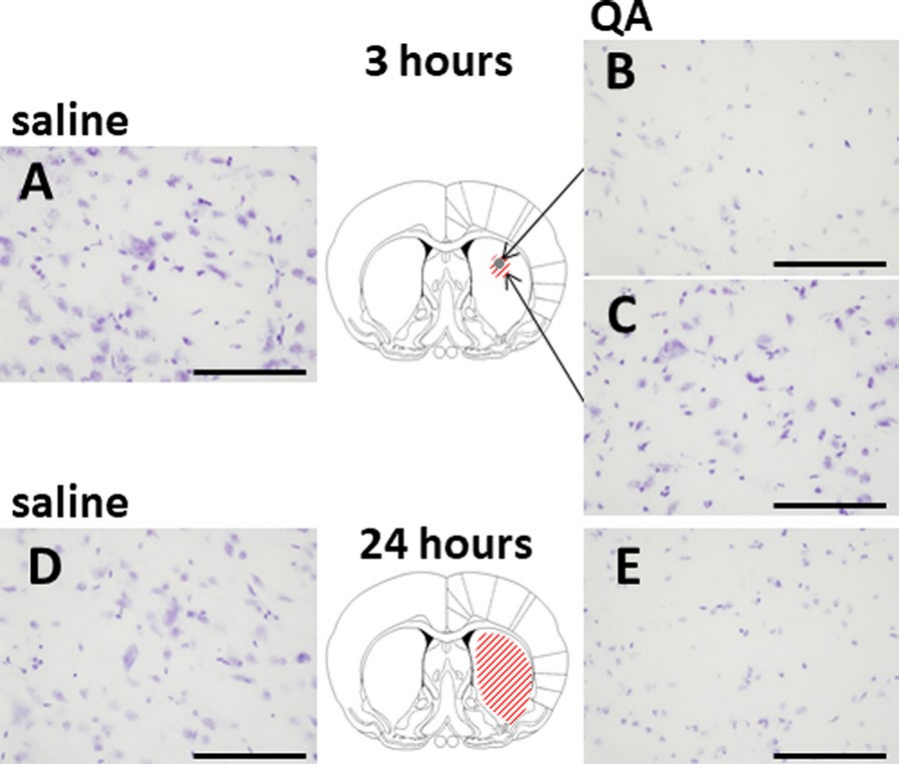
Nissl-stained brain slices within the striatum after the acquisition of ex vivo fluorescent images. QA was injected into the striatum, and saline was simultaneously injected into the contralateral striatum. Three hours (**A**–**C**) or 24 h (**D** and **E**) after QA or saline injection. Neuronal degradation was observed in the greyed-out area within an approximately 1.0-mm-diameter area (**B**) and in the whole striatum (**E**). The red hatched area corresponds to the area of increased fluorescence signal. The images in A, C, and D showed no abnormalities. Bar = 100 µm

Nissl staining of brain slices was performed after the acquisition of ex vivo fluorescent images, 3 h after QA injection, which showed remarkable neuronal degradation in the striatum at the site of QA injection (Fig. 5B), although in a narrower area than the area associated with a strong fluorescence signal (Fig. 2A). This neuronal degradation was localised within an approximately 1.0-mm-diameter area (Fig. 5B), and neurons a few mm away from the injection site remained intact (Fig. 5C). One day after QA injection, the neuronal degradation had spread throughout the entire striatum (Fig. 5E), but no abnormalities were observed in the prefrontal cortex (Additional file 1: Fig. 1B) and thalamus (Additional file 1: Fig. 1D).

**Fig. 6:**
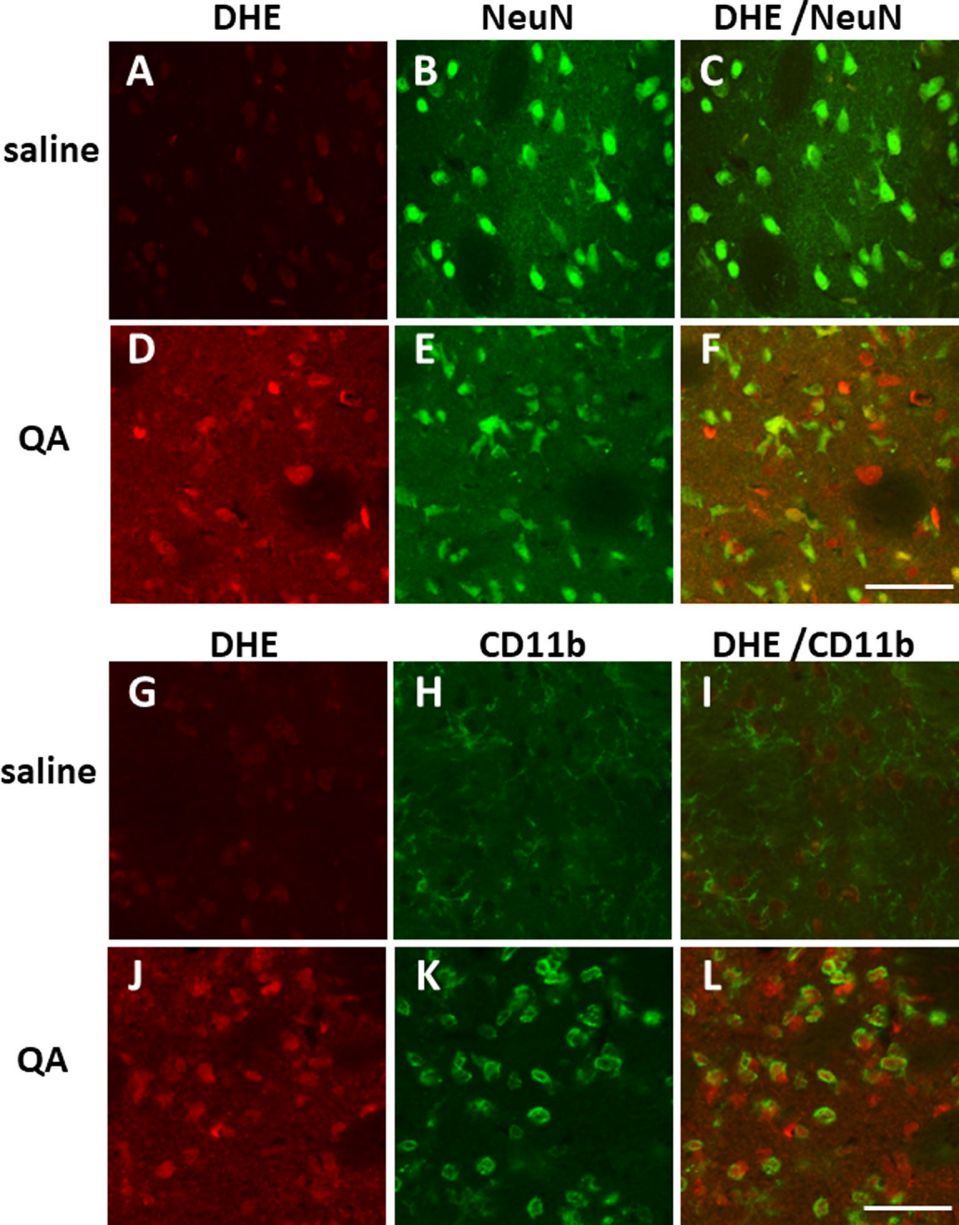
Immunohistochemical analysis of NeuN- or CD11b-positive cells in the saline- or QA-injected rat striatum. Saline or QA was injected into the striatum 24 h before DHE administration. Perfusion fixation for immunohistochemical study was started 1 h after DHE administration. Bar = 50 µm

To clarify which cell types were producing DHE-derived fluorescence signals, immunohistochemical studies were performed in the striatum 24 h after QA injection, using monoclonal antibodies against NeuN, a marker for neurons (Fig. 6A–F), and CD11b, a marker for activated microglia (Fig. 6G–L). In the QA-injected striatum, the quantity of NeuN-positive cells was reduced to 60 ± 9% (Fig. 6E, 505 ± 122 cells/mm^2^, n = 4, p < 0.01) compared with that in the saline-injected striatum (Fig. 6B, 827 ± 122 cells/mm^2^, n = 4), and CD11b-positive cells were increased to 591 ± 165% (Fig. 6 K, 531 ± 38 cells/mm^2^, n = 4, p < 0.001) compared with those in the saline-injected striatum (Fig. 6H, 96 ± 22 cells/mm^2^, n = 4). Furthermore, as shown in Fig. 6L, the majority of DHE fluorescence signals colocalised with CD11b-positive cells in the QA-injected striatum.

## Discussion

QA is an endogenous analogue of glutamate that acts as an NMDA receptor agonist [25, 26]. QA is an excitotoxic metabolite of the kynurenine pathway with high in vivo potency as a neurotoxin. QA administration increases the levels of extracellular glutamate [35, 36] and increases cytosolic Ca^2+^ concentrations in both neurons and astrocytes. Furthermore, QA excitotoxicity is associated with its ability to increase lipid peroxidation [37] and induce free radical generation [38], which consequently induces oxidative stress and energetic dysfunction. Intrastriatal QA injections induce excitotoxicity in animals [39], resulting in neuronal lesions [28].

In the present study, increased ROS generation, as imaged using a fluorescent signal, was detected in a focal area within the striatum as early as 3 h after QA injection, and this signal spread throughout the striatum and parts of the cerebral cortex 24-h post-injection. At 3 h after QA injection, the area of neuronal degradation in the striatum was observed to be smaller than the area of excessive ROS generation. At 24 h after QA injection, the ROS-associated fluorescent signal was primarily detected in microglia in the QA-injected striatum. Although ^18^F-BCPP-EF uptake was unchanged 3 h after QA injection, a marked decrease in uptake was observed after 24 h, not only in the striatum but also in ​​the cerebral hemisphere, indicating that the delayed impairment of MC-I activity was more extensive than ROS generation 24 h after QA injection.

The focal ROS generation observed 3 h after QA injection may reflect the mitochondrial ROS generation induced by excessive Ca^2+^ influx, mediated by NMDA receptor activation or represent ROS generated by the Fenton reaction between QA-Fe^2+^ complexes [40]. In our preliminary study, a similar degree of focal ROS generation was observed as early as 1 h after QA injection (data not shown). Santamaría et al. have shown that transient increases in ROS generation associated with the overactivation of NMDA receptors and other mechanisms occur as early as 1 h after QA infusion into the rat striatum in vivo [41]. Although several other reports have shown that QA injections modify ROS generation at later time points after QA injections into animal brains [38, 42], a large amount of ROS generation appears to occur in a restricted area, and a mild increase in ROS generation appears to occur throughout the striatum.

The present microscopic analyses of combined fluorescence signals and immunohistochemical staining demonstrated that explosive ROS generation occurred in microglia located within the striatum 24 h after QA injection. At this time point, neuronal degradation and microglial activation were detected in the striatum. Neuronal damage caused by excitotoxic factors has been reported to stimulate a proinflammatory cascade. In this cascade, microglial activation occurs in response to neuronal excitotoxic injury, which, in turn, activates microglia to release ROS and neurotoxic cytokines [43–45]. This cascade results in a self-propelled, progressive cycle of microglial activation and neuronal damage. Under these conditions, excessive ROS generation in the microglia may lead to an oxidant/antioxidant imbalance, in which the antioxidative response may not be sufficient to normalise redox homeostasis. The ROS generation by microglia can be originated from several sources; however, NADPH oxidase activation has been reported to enhance the release of other proinflammatory factors from microglia [46, 47], and this enzyme might represent an important factor in microglia-mediated neurotoxicity. Oxidative stress is well known to be associated with inflammation and disease onset; thus, ROS generation in microglia may serve as a valuable target with therapeutic benefits for the treatment of neuronal disorders.

In the present study, ^18^F-BCPP-EF uptake in the QA-injected rat brain was unchanged after 3 h, indicating that MC-I activity remained intact during this early period after QA injection. Bordelon et al. reported progressive mitochondrial dysfunction in the rat striatum 12 h after QA injection [48]. Ribeiro et al. also reported reduced activity in MC-II and MC-III in the respiratory chain in the striatum 12 h after QA injection but not 3 or 6 h after QA injection [49]. These results, together with those of the present study, indicate that mitochondrial proteins or their functions may survive through at least the early period after QA-induced excitotoxic injury.

A decrease in MC-I activity 24 h after QA injection could be observed not only in the striatum, where neuronal degradation occurred, but also in the cerebral hemisphere, where cells remained intact. In the QA-injected striatum, decreased MC-I activity may reflect mitochondrial dysfunction in the proinflammatory cascade and the self-propelled, progressive cycle of microglial activation and neuronal degradation. Furthermore, the inhibition of MC-I has been reported to induce robust ROS generation from the mitochondria, which activates NLR family pyrin domain-containing 3 inflammasome [50, 51], driving several inflammatory processes and mediating the release of interleukin-1 family cytokines [52]. Thus, ^18^F-BCPP-EF appears to serve as an excellent probe that reflects neuronal mitochondrial dysfunction during the proinflammatory cascade.

MC-I activity, as measured using ^18^F-BCPP-EF, was reduced in areas where cells remained intact 24 h after QA injection. Brain damage often causes changes in cellular metabolism and function, not only near the neural degradation area but also in more remote brain regions that are functionally and anatomically connected to the injured area. When QA is injected into the striatum of adult rats, neurogenesis has been reported to occur between 1 and 14 days after injection [53, 54]. One possibility is that neurogenesis shifts the balance of energy production from mitochondria-related oxidative metabolism to glycolysis in the cerebral hemisphere on the QA-injected side. Alternatively, the inhibition of MC-I activity has been reported to activate microglia [55–57]. Another study suggested that mitochondrial dysfunction in microglia exacerbated the proinflammatory M1 phenotype and increased ROS production [58]. In addition, the removal of impaired or dysfunctional mitochondria has been reported to be neuroprotective [59]. Thus, QA injection into the striatum has been speculated to stimulate neurogenesis or cause more severe and extensive damage to the cerebral hemisphere on the QA-injected side during later phases. Based on the results of the present study, whether the observed decrease in MC-I activity is temporary due to a defensive cellular response or is long-lasting, associated with subsequent cellular damage, is not known. Whether reduced MC-I activity counteracts or exacerbates pathological effects is also unclear. These questions are important subjects for future research.

In this study, multiple molecular probes were used to image and monitor the intracellular neurodegenerative processes. This approach is useful for understanding brain function and disease and could contribute to disease diagnosis, therapeutic development, and the assessment of therapeutic effects. In the brain, functional imaging, including neuron-microglia, or neuron-astrocyte interactions, is also necessary. In the present study, we used ex vivo imaging to perform ROS detection. For in vivo imaging, we recently developed a novel PET probe, ^18^F-labelled dihydromethidine, to detect ROS [60]. Multi-tracer imaging in the same animals using PET probes is expected in future studies.

## Conclusion

We successfully obtained functional images of focal ROS generation during the early period of excitotoxic injury, and microglial ROS generation and mitochondrial dysfunction were observed during the progression of the inflammatory response. Both ex vivo DHE imaging and in vivo ^18^F-BCPP-EF-PET were sufficiently sensitive to detect the respective processes of QA-induced brain damage. Our study contributes to the functional imaging toolbox available to monitor multiple events in the pathological process.

## Supplementary Information

**Additional file 1. Fig. 1** Nissl-stained brain slices in the prefrontal cortex (A and B) and thalamus (C and D) after the acquisition of ex vivo fluorescent images. The day before, QA was injected into the striatum, and saline was simultaneously injected into the contralateral striatum. No abnormalities were observed following QA injection. Bar = 100 µm.

## Data Availability

The data that support the findings of this study are available from the corresponding author, R-hosoi, upon reasonable request.
