# Supplementary figures and images for: Evaluation of intracellular processes in quinolinic acid-induced brain damage by imaging reactive oxygen species generation and mitochondrial complex I activity
Source: EJNMMI Res. 2021 Oct 9;11:99. doi: 10.1186/s13550-021-00841-3 (PMC8502189; doi:10.1186/s13550-021-00841-3)

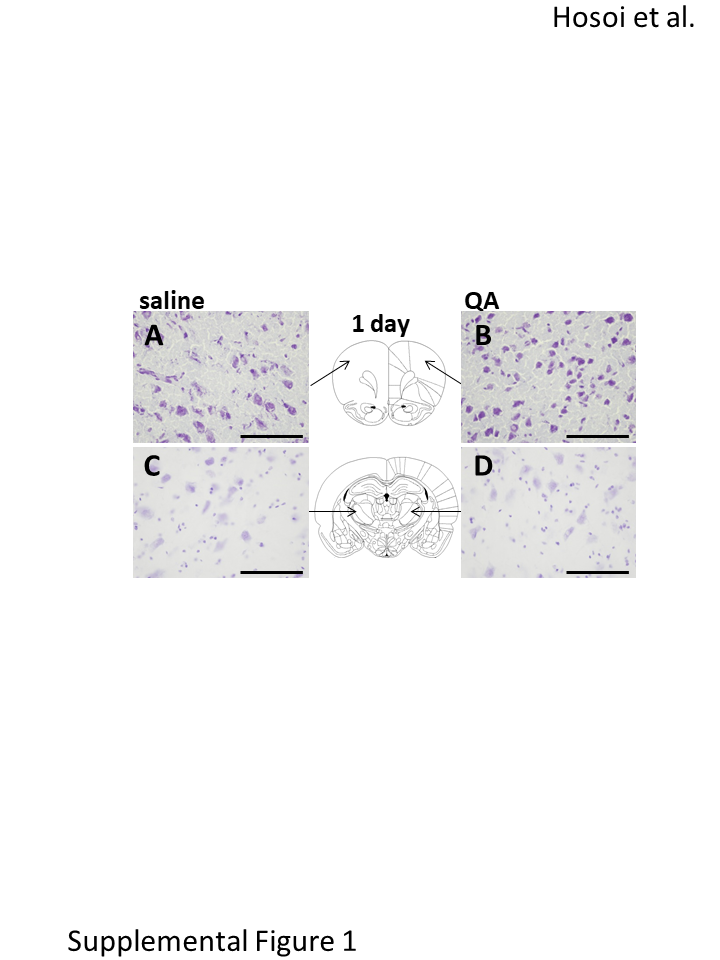

Supplement: Supplementary file 1 — Additional file 1. Fig. 1 Nissl-stained brain slices in the prefrontal cortex (A and B) and thalamus (C and D) after the acquisition of ex vivo fluorescent images. The day before, QA was injected into the striatum, and saline was simultaneously injected into the contralateral striatum. No abnormalities were observed following QA injection. Bar = 100 µm. [file 13550_2021_841_MOESM1_ESM.tif]
